# Mutational Landscape Analysis of BRCA1/2 and Identification of Extracellular-Vesicle-Related Biomarkers in Triple-Negative Breast Cancer

**DOI:** 10.3390/biomedicines14010178

**Published:** 2026-01-14

**Authors:** Yuqiu Hu, Jiali Wu, Lu Sun, Zishan Xie, Ming Li, Lu Yuan, Rui Huang, Weixing Zhang

**Affiliations:** Department of Breast Surgery, The Eighth Affiliated Hospital of Sun Yat-sen University, 3025# Shennan Road, Shenzhen 518000, China

**Keywords:** triple-negative breast cancer, *BRCA1/2*, extracellular vesicles, *PLA2G5*

## Abstract

**Background**: Triple-negative breast cancer (TNBC), defined by the absence of estrogen receptor (ER), progesterone receptor (PR), and human epidermal growth factor receptor 2 (HER-2) expression, is associated with increased *BRCA1/2* mutation rates. Extracellular vesicles (EVs) play a pivotal role in TNBC progression. This study aimed to analyze *BRCA1/2* mutations and identify EV-related biomarkers for TNBC by employing TNBC-related datasets and EV-related genes (EVRGs). **Methods**: Initially, *BRCA1/2* mutations in TNBC patients were examined. Differentially expressed EVRGs (DE-EVRGs) were identified by integrating the results of both differential expression analysis and weighted gene co-expression network analysis (WGCNA). Biomarkers were identified using Receiver Operating Characteristic (ROC) and Kaplan–Meier (K–M) analyses. Finally, functional enrichment, drug prediction, molecular docking, and reverse transcription quantitative polymerase chain reaction (RT-qPCR) analyses were performed. **Results**: Waterfall plots indicated that *TP53* exhibited the highest mutation frequency in both the mutation (MUT) and wild-type (WT) group. Four distinct types of immune cells (for example, eosinophils and neutrophils) showed significantly elevated expression levels in the WT group. Notably, *PLA2G5* was identified as a biomarker of TNBC and its expression was significantly lower in TNBC (*p* = 0.0025). Functional analysis demonstrated that *PLA2G5* is enriched in the “drug metabolism cytochrome P450” pathway. Finally, 20 drugs targeting *PLA2G5* were identified, among which leukotriene C4 demonstrated a binding affinity of −7.2 kcal/mol. This finding suggests that leukotriene C4 has potential therapeutic applications for the treatment of TNBC. **Conclusions**: Our study found significant differences between the MUT and WT groups, identifying *PLA2G5* as a biomarker for TNBC and offering a theoretical basis for TNBC treatment.

## 1. Introduction

Breast cancer (BC) is the most common cancer in women all over the world and was the second leading cause of cancer-related deaths in 2020 [1]. Triple-negative BC (TNBC) is characterized by a lack of the estrogen receptor (ER), progesterone receptor (PR), and human epidermal growth factor receptor 2 (HER-2), accounting for 10–15% of BC cases. TNBC is an aggressive BC with a high recurrence rate, early metastatic spread, and poor survival outcomes. Current treatment options for TNBC are much more limited than for other subtypes with respect to the lack of effective targeted therapies, such as endocrine therapy and anti-HER-2 targeted therapy. Surgery and chemotherapy remain the first-line therapy for TNBC [2,3]. Chemotherapy is the cornerstone treatment for TNBC, anthracyclines, cyclophosphamide, and taxane regimens serving as basic therapeutic schedules in early TNBC patients. However, the rate of complete pathologic response (PCR) in patients receiving conventional neoadjuvant chemotherapy regimens is only 35–45%, and the recurrence rate is up–20–40% in the first two years [4]. Therefore, there is an urgent need to find new therapeutic targets or prognostic biomarkers.

BC susceptibility gene 1 or 2 (*BRCA1/2*) proteins, which are deoxyribonucleic acid (DNA) double-strand damage repair proteins encoded by BRCA genes, play an important role in maintaining genomic stability by participating in error-free homologous DNA repair (HDR) when a DNA double-strand break (DSB) occurs [5]. BRCA1/2 mutations are associated with an elevated lifetime risk of multiple cancers, including BC, ovarian cancer, prostatic cancer and pancreatic cancer. Patients with BRCA1/2 mutations account for about 5–10% of all BC patients and 3.9% of Chinese BC patients [6]. The mutation rate of *BRCA1/2* in TNBC patients is 15–20%, higher than that in other BC subtypes [7]. Current studies indicate that TNBC patients with *BRCA1/2* mutations can benefit from platinum-type neoadjuvant or adjuvant chemotherapy [8,9]. Poly-ADP-ribosyl polymerase inhibitors (PARPi) are considered a first-line treatment option for advanced triple-negative BC with gBRCA mutations and PD-L1 negative [10]. Therefore, exploration of TNBC with *BRCA1/2* mutations is helpful to further improve the effectiveness of treatment strategies for patients with TNBC.

Extracellular vesicles (EVs) are membrane-bound vesicles secreted from multiple cell types [11]. In recent years, EVs have received considerable attention as a novel cell-cell communication mechanism, especially in the field of cancer. EVs are complex carriers of intercellular communication and are thought to affect macrophage activation [12]. Studies have shown that CSF-1-containing EVs released from TNBC promote the formation of tumor immune microenvironment related to a good prognosis of TNBC patients. EVs and soluble molecules released by TNBCs promote the differentiation of monocytes into different macrophage fates. EVs specifically promoted pro-inflammatory macrophages with interferon response characteristics. It has been proven that EVs contribute to TNBC angiogenesis, immune escape, tumor proliferation, drug resistance and distant metastasis [13].

In this study, we explored mutational landscape differences in TNBC patients with or without *BRCA1/2* mutations based on the The Cancer Genome Atlas (TCGA) database. In addition, EV biomarkers related to TNBC and *BRCA1/2* mutations were screened and identified using bioinformatics methods, and the biological pathways and regulatory network relationships involving the biomarkers were obtained.

## 2. Materials and Methods

### 2.1. Data Collection

The Cancer Genome Atlas-BC (TCGA-BC) dataset, which included expression matrices, clinical information, survival data, and somatic mutation information for BRCA samples, was downloaded from the TCGA database (https://portal.gdc.cancer.gov/, accessed on 19 January 2024). The samples were then screened based on the criteria of ER, PR, and human epidermal growth factor receptor 2 (Her-2) negativity to obtain TCGA-TNBC cohort. This cohort included 118 TNBC tumor tissue samples and 99 control breast tissue samples. Similarly, 320 TNBC samples with complete survival information were retrieved from the cBioPortal database (https://www.cbioportal.org/, accessed on 25 January 2024) as a validation cohort. In total, 25 extracellular vesicle-related genes (EVRGs) were obtained from the “extracellular vesicle biogenesis” Gene Ontology Biological Process (GO-BP) pathways from the Molecular Signatures Database (MSigDB). The EVRGs are listed in Supplementary Table S1.

### 2.2. Mutational Landscape Analysis of BRCA1/2

Mutation and copy number variation data of BRCA1/2 in the TCGA-TNBC cohort were downloaded from cBioPortal. Patients were divided into two groups: the mutation (MUT) (with *BRCA1/2* mutations) and the wild-type (WT) group (without *BRCA1/2* mutations). Furthermore, the clinical characteristics of the MUT and WT groups was explored, including stage (I-IV), race (White, African, Asian), pathologic M (M0, MX, M1), N (N0–N3), and T (T1–T4, TX).

### 2.3. Difference Analysis and Mutation Spectrum Assessment

Homologous Recombination Deficiency (HRD), Loss of Heterozygosity (LOH), Telomeric Allelic Imbalance (TAI), and Large-Scale Transition (LST) scores were found to be closely associated with *BRCA1/2* mutations. Consequently, these scores were used to investigate differences between the MUT and WT groups. Initially, these scores were obtained from literature [14]. Subsequently, a Wilcoxon test was conducted to compare the differences in these four scores between the MUT and WT groups (*p* < 0.05).

Functional enrichment analysis was performed to explore the differences in biological processes between the MUT and WT groups. The reference gene set “h.all.v2023.2. Hs.symbols.gmt” were acquired from the MSigDB database. In the TCGA-TNBC cohort, differential expression analysis (MUT vs. WT) was conducted using “DESeq2” (v 1.42.0) [15] and the log_2_FoldChange (FC) was calculated and ranked from highest to lowest. Subsequently, “clusterProfiler” (v 4.10.0) [16] was utilized to perform Gene Set Enrichment Analysis (GSEA) with a significance threshold of *p* < 0.05, and a False Discovery Rate (FDR) < 0.05.

Moreover, somatic mutation data for each sample in the MUT and WT groups were obtained to investigate the potential genomic mutation between the MUT and WT groups. Then, “Maftools” (v 2.17.10) [17] was employed to yield the waterfall plots presenting the top 20 most frequently mutated genes in MUT and WT groups, respectively. Additionally, “Maftools” (v 2.17.10) was utilized to calculate the Tumor Mutational Burden (TMB) score for MUT and WT groups. Wilcoxon test was employed to explore the differences of TMB score between MUT and WT groups (*p* < 0.05).

### 2.4. Immune Microenvironment Analysis

To analyze the differences in the immune microenvironment between the MUT and WT groups, the single-sample GSEA (ssGSEA) algorithm was employed to calculate the infiltration proportions of 28 immune cell types in samples from both groups. The results were displayed as a heatmap using the “ComplexHeatmap” package (v 2.16.0) [18]. Subsequently, the differences in infiltration proportions were analyzed using the Wilcoxon test, with the significance threshold set at *p* < 0.05. Differences were further visualized in a boxplot via the “ggplot2” package (v 3.4.4) [19].

In the tumor microenvironment (TME), the tumor immune cycle consists of seven critical steps: release of tumor antigens, tumor antigen presentation, T cell activation, T cell migration into tumor tissue, T cell infiltration into tumor tissue, recognition of tumor cells by T cells, and clearance of tumor cells. To analyze the differences in anticancer immune status between the MUT and WT groups, we used the Tracking Tumor Immunophenotype (TIP) tool (http://biocc.hrbmu.edu.cn/TIP/analysis.jsp, accessed on 20 January 2024) to calculate scores for each of these seven steps separately in samples from both groups. Subsequently, the scores for each step between the WT and MUT groups were compared using the Wilcoxon test (*p* < 0.05) and the results were visualized using the “ggplot2” package.

In addition, to evaluate the immune microenvironment in TNBC patients, Estimation of STromal and IMmunAl cells in Tumors using Expression (ESTIMATE) algorithm was executed using the “estimate” package (v 1.0.13) to compute three types of scores: stromal score, immune score, and ESTIMATE score. Wilcoxon test was performed to investigate differences in these scores between MUT and WT groups (*p* < 0.05).

### 2.5. Drug Sensitivity Analysis

To evaluate the sensitivity of TNBC patients in the MUT and WT groups to conventional drugs, data for 198 drugs were acquired from the Genomics of Drug Sensitivity in Cancer database (GDSC, https://www.cancerrxgene.org/, accessed on 19 January 2024). The inhibitory concentration 50 (IC_50_) values for each drug were calculated using the “oncoPredict” package (v 0.2) [20]. Subsequently, the differential IC_50_ values between the MUT and WT groups were compared using Wilcoxon test (*p* < 0.05).

### 2.6. Analysis of Differences in Immune Infiltration, Anti-Tumor Immune Status and Drug Sensitivity Stratified Based on Key Clinical Features

To verify the robustness of core differences, exclude interference from clinical feature heterogeneity, and explore subgroup-specific differences, this study stratified the TCGA-TNBC cohort by age, race, cancer stage, menopausal status, radiotherapy status, and targeted therapy status (hierarchical information is shown in Supplementary Table S2). Within each subgroup, the R package “GSVA” (v1.48.2) [21] was used with the ssGSEA algorithm to analyze the infiltration of 28 immune cell types. The TIP tool (http://biocc.hrbmu.edu.cn/TIP/analysis.jsp, accessed on 20 January 2024) was employed to evaluate tumor immune cycle scores, and the Wilcoxon test was applied to compare the IC_50_ values of nine drugs. A *p*-value < 0.05 was considered statistically significant. All statistical analyses were performed using R (version 4.2.3).

### 2.7. Differential Expression Analysis

In the TCGA-TNBC cohort, two distinct sets of differentially expressed genes (DEGs1 and DEGs2) were identified using the “DEseq2” package, based on the criteria of *p* < 0.05 and |log_2_FC| > 0.5. Specifically, DEGs1 were identified by comparing TNBC samples with control samples, whereas DEGs2 were identified by comparing MUT samples with WT samples. To visually represent these findings, volcano plots were generated using “ggplot2” and heat maps were constructed using “circlize” (v 0.4.15) [22] to visualize the top 20 upregulated and downregulated genes.

### 2.8. Identification and Function Analysis of Differentially Expressed EVRGs (DE-EVRGs)

Based on the expression of EVRGs in the MUT and WT samples of the TCGA-TNBC cohort, EVRG scores were calculated for all samples using the ssGSEA algorithm implemented in the “GSVA” package (v 1.49.4) [21]. Subsequently, differences in EVRG scores between MUT and WT samples were evaluated using Wilcoxon test, with statistical significance set at *p* < 0.05. To explore the genes closely associated with EVRGs scores, the Weighted Gene Co-expression Network Analysis (WGCNA) approach was applied using the “WGCNA” package (v 1.72-5) [23]. WGCNA is a systems biology method designed to construct gene co-expression networks, identify modules of highly interconnected genes, and relate these modules to sample traits or clinical features. Initially, all TNBC samples in the MUT and WT groups were clustered to identify and remove the outliers. Then, the soft threshold (power) was selected such that the scale-free R2 for the first time was more than 0.9, and the mean connectivity was close to 0. Subsequently, a gene co-expression matrix was built, and modules of co-expressed genes were identified using hierarchical clustering, with the minimum module size set at 400 genes. Subsequently, to identify the modules most correlated with EVRG scores, the Spearman correlation coefficient was calculated between the modules and the EVRG scores (|correlation (cor)| > 0.3, *p* < 0.05). Notably, the module showing the highest significant related to EVRGs scores was selected as the key module, and the genes within this key module were designated as key module genes.

Later, to identify DE-EVRGs, the sets of DEGs1, DEGs2, and key module genes overlapped, highlighting genes that were consistently differentially expressed across these analyses. To further explore the function of DE-EVRGs, Gene Ontology (GO) and Kyoto Encyclopedia of Genes and Genomes (KEGG) analyses were performed using the “clusterProfiler” package (*p* < 0.05). The GO system encompassed three aspects: biological processes (BPs), molecular functions (MFs), and cellular components (CCs).

### 2.9. Identification of Biomarkers

In the TCGA-TNBC cohort, we evaluated the discriminative ability of DE-EVRGs and identified candidate genes 1 and 2 to distinguish between the MUT and WT groups, as well as between TNBC and control samples. Receiver Operating Characteristic (ROC) curves were generated using the “pROC” package (v 1.18.4) [24]. Genes with an area under the curve (AUC) value greater than 0.7 were selected as candidate genes 1 and candidate genes 2, respectively. In addition, to further evaluate the association between DE-EVRGs and TNBC patient survival, TNBC patients from the TCGA-TNBC cohort were grouped into high and low-expression groups using the optimum cutoff value of DE-EVRGs expression levels calculated by the ‘surv cutpoint’ function. Kaplan–Meier (K–M) survival analysis was executed via the log-rank test from the “survminer” package (v 0.4.9) [25] to assess the survival differences between the two expression groups. Genes with *p* < 0.05 were identified as candidate genes 3.

Key genes were determined by overlapping candidate genes 1, 2, and 3. To validate these key genes, a validation cohort consisting of 320 TNBC samples was stratified into high and low expression groups based on the optimal threshold of DE-EVRGs expression. The log-rank test was subsequently employed to evaluate the survival differences between these groups. Key genes that showed significant differences in the KM curves (*p* < 0.05) were considered biomarkers.

### 2.10. Verification of the Independent Prognostic Value of Biomarkers and Correction Analysis of Clinical Confounding Factors

To further verify the independent prognostic value of the biomarker PLA2G5, the R package “survival” was used. Univariate Cox regression analysis of PLA2G5 was conducted in the TCGA dataset through the “survival” package (HR ≠ 1, *p* < 0.1) [26]. The Schoenfeld residual test was used to verify whether the variables met the proportional hazards (PH) assumption (*p* > 0.05); The significant variables screened by the single factor were then incorporated into the multivariate Cox regression model. After correcting for the confounding effects of clinical variables, the independent prognostic value of PLA2G5 was re-evaluated, and the overall PH hypothesis of the model was verified at the same time. Finally, the HR, 90% CI and *p*-value of the regression analysis are presented through a forest plot, and the PH test results are shown in a Schoenfeld residual trend chart.

### 2.11. Function Analysis of Biomarkers

GeneMANIA was adopted to identify genes functionally linked to biomarkers, and a gene-gene interaction (GGI) network was built. Functions with an FDR < 0.05 are highlighted with distinct colors, with each color corresponding to a specific biological function. Furthermore, for a more comprehensive understanding of the biomarkers, functional enrichment analyses were performed on MUT and WT samples, as well as TNBC and control samples, within the TCGA-TNBC cohort. Initially, the correlation coefficients of gene expression relative to biomarkers were calculated and ranked. The background gene set “c2.cp.kegg.v2023.1. Hs.symbols.gmt” were obtained from the MSigDB database (https://www.gsea-msigdb.org/gsea/msigdb, accessed on 25 January 2024). Subsequently, GSEA was conducted on MUT and WT samples, as well as TNBC and control samples, using the “clusterProfiler” package (*p* < 0.05).

### 2.12. Subcellular and Chromosomal Localization Analyses

To explore the distribution of biomarkers within subcells, database prediction was performed using the mRNALocater database (http://bio-bigdatacn/mRNALocater, accessed on 25 January 2024) to determine their subcellular distribution across the cytoplasm, endoplasmic reticulum, extracellular region, mitochondria, and nucleus.

Additionally, to determine the chromosomal locations of the biomarkers, the “RCircos” package (v 1.2.2) [27] was used for visualization.

### 2.13. Regulation Network Analysis

We utilized the mirTarbase (http://mirtarbase.cuhk.edu.cn, accessed on 25 January 2024) and TarBase (https://dianalab.e-ce.uth.gr/tarbasev9, accessed on 25 January 2024) databases to predict miRNAs targeting these biomarkers via the NetworkAnalyst platform. Subsequently, we employed the starBase and miRNet databases to predict Long non-coding riboNucleic acids (lncRNAs) targeting these key micro riboNucleic acids (miRNAs). Similarly, we integrated both databases to predict key lncRNAs. Finally, we constructed and visualized the complete lncRNA-miRNA-mRNA regulatory network using Cytoscape (v.3.9.1) [28].

Additionally, researchers predicted transcription factors (TFs) targeting biomarkers using hTFtarget data (https://guolab.wchscu.cn/hTFtarget/#!, accessed on 25 January 2024) and constructed TF-mRNA networks with Cytoscape.

### 2.14. Drug Prediction and Molecular Docking Analyses

To probe potential drugs for the treatment of TNBC based on the identified biomarkers, the “enrichR” package (v 3.2) was utilized (*p* < 0.05). A drug-biomarker network was built and visualized using Cytoscape (v 3.9.1). Subsequently, to evaluate the binding affinity between the drug and the biomarkers, the drug with the highest interaction scores for the biomarker was chosen for molecular docking studies. The 3D molecular structures of the drugs (acting as ligands) were sourced from the PubChem database, while the protein crystal conformations of the biomarkers (acting as receptors) were obtained from the UniProt database. Molecular docking analyses were performed by CB-Dock2, and the binding energy was calculated. A binding energy of less than −5 kcal/mol typically indicates a strong binding affinity between the drug and the biomarker, suggesting the potential for effective molecular interaction.

### 2.15. Reverse Transcription Quantitative Polymerase Chain Reaction (RT-qPCR)

Ten tissue samples (five TNBC tissues and five matched adjacent normal tissues) were acquired from the clinic at the Eighth Affiliated Hospital, Sun Yat-sen University. All the participants informed consent. The study was approved by the Ethics Committee of the Eighth Affiliated Hospital, Sun Yat-sen University (approval number: 2024-302-02). Total RNA was isolated using TRIzol reagent (Invitrogen, Waltham, MA, USA) according to the manufacturer’s instructions, and RNA concentration was measured using a NanoPhotometer N50 spectrophotometer (Implen, Munich, Germany). cDNA was synthesized via reverse transcription using the SureScript First-Strand cDNA Synthesis Kit. Primer sequences are listed in Supplementary Table S3. OCR detection was performed using a CFX Connect real-time PCR system (Bio-Rad, Hercules, CA, USA). Relative mRNA quantification was measured via the 2^−△△Ct^ method.

### 2.16. Statistical Analysis

All statistical analyses were executed via R (version 4.2.3). * *p* < 0.05, unless otherwise stated.

## 3. Results

### 3.1. The MUT and WT Groups Exhibited Significant Differences

**Figure 1:**
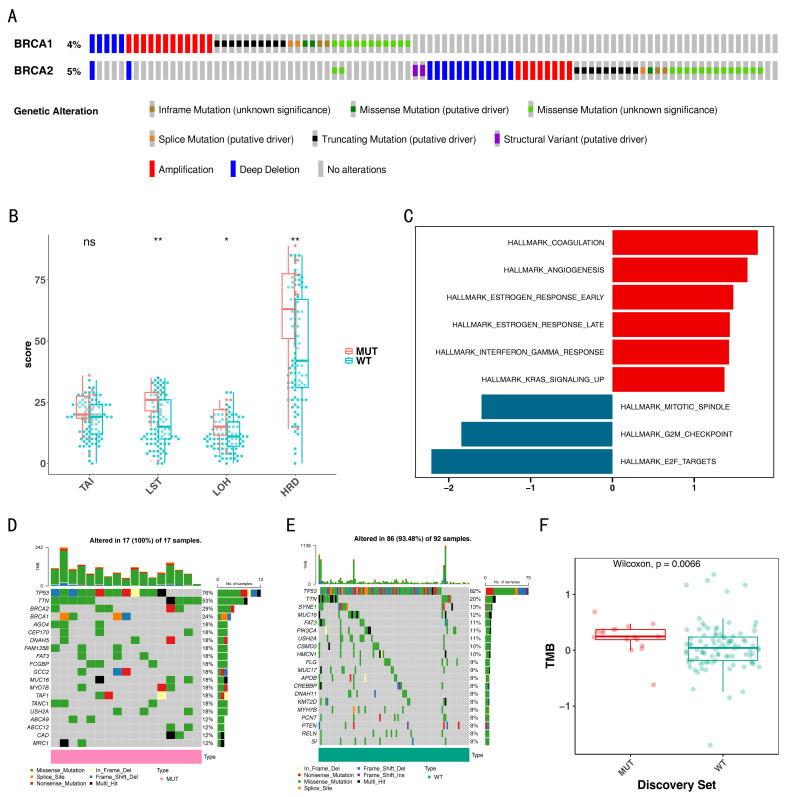
The mutation (MUT) and wild-type (WT) group groups exhibited significant differences. (**A**) The mutation rates of *BRCA1* and *BRCA2* differ between MUT and WT groups. (**B**) Differences between MUT and WT groups in telomeric allelic imbalance (TAI), large-scale transitions (LST), loss of heterozygosity (LOH), and homologous recombination deficiency (HRD) scores. ns indicates not significant, * indicates *p* < 0.05, ** indicates *p* < 0.01. (**C**) Biological function analysis identified nine significantly enriched pathways between MUT and WT group. Waterfall plots showed the 20 most frequently muatated genes in MUT group (**D**) and WT group (**E**). (**F**) MUT groups had higher tumor mutational burden (TMB) scores compared to WT groups.

In the TCGA-TNBC cohort, the mutation rates for *BRCA1* (4%) and *BRCA2* (5%) were illustrated, showing genetic alterations, such as amplifications, deep deletions, and other variations (Figure 1A). Table 1 further detailed the distribution of clinical features between the MUT (19 TNBC patients) and WT (99 TNBC patients) groups. Both groups had a higher representation of stage II, White race, pathological M0, and N0. Additionally, regarding pathologic T, the MUT group had a greater proportion in T2 (78.9%), whereas the WT group had a higher proportion in T1 (64.6%).

Moreover, significant differences in large-scale transitions (LST), loss of heterozygosity (LOH), and homologous recombination deficiency (HRD) scores were observed between the MUT and WT groups (*p* < 0.05), with the MUT group exhibiting higher scores of LST, LOH, and HRD. However, no significant difference was noted in the telomeric allelic imbalance (TAI) scores (Figure 1B). Biological function analysis between the MUT and WT groups identified nine significantly enriched pathways (*p* < 0.05, FDR < 0.05), including “Hallmark Coagulation”, “Hallmark Angiogenesis”, “Hallmark E2F Targets”, “Hallmark G2M Checkpoint”, etc. (Figure 1C). Additionally, waterfall plots depicting the MUT and WT groups showed that *TP53* had the highest mutation frequency in both groups, with 76% in the MUT group (17 samples with genetic mutations) and 82% in the WT group (92 samples with genetic mutations) (Figure 1D,E). The types of mutations include missense and nonsense mutations. Additionally, there was a marked difference in the TMB scores between the MUT and WT (*p* = 0.0066). Patients with mutations exhibited significantly higher TMB scores compared to those in the WT (Figure 1F).

### 3.2. Impact of Immunotherapy and Drug Sensitivity on BRCA1/2 Mutation in TNBC

**Figure 2:**
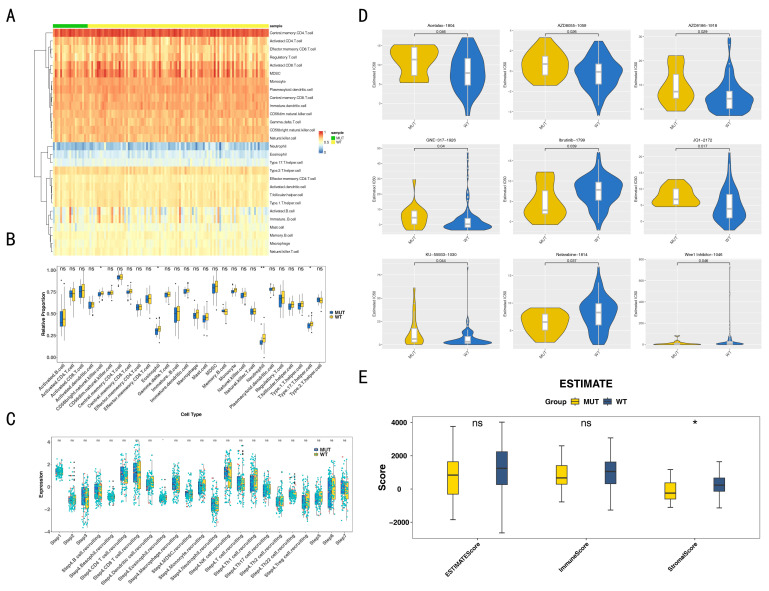
Immunotherapy and drug sensitivity on *BRCA1/2* mutation in triple-negative breast cancer (TNBC). (**A**) Infiltration proportions of 28 immune cells in mutation (MUT) and wild-type (WT) group are shown with a heatmap (**A**) and box plot (**B**). ns indicates not significant, * indicates *p* < 0.05, ** indicates *p* < 0.01. (**C**) The differences in anticancer immune status between the MUT and WT groups. (**D**) Drug sensitivity differences between the MUT and WT groups. (**E**) WT groups had a higher stromal score compared to MUT groups. ns indicates not significant, * indicates *p* < 0.05.

Figure 2A depicts the infiltration proportions of 28 immune cells in both the MUT and WT groups. Notably, four differential immune cells exhibited significantly higher expression in the WT group: CD56 bright natural killer cells, eosinophils, neutrophils, and type 17 T helper cells (Figure 2B). Additionally, Figure 2C demonstrated a significant difference in Step 4 eosinophil recruiting between the MUT and WT groups, with the WT group showing a higher score in Step 4 eosinophil recruiting.

Furthermore, drug sensitivity analysis revealed conspicuous differences in the IC_50_ values of the nine drugs between the MUT and WT groups (*p* < 0.05) (Figure 2D). Among them, Acetalax 1804, AZD8055 1059, AZD8186 1918, GNE-317 1926, JQ1 2172, and KU-55933 1030 exhibited higher IC_50_ values in the MUT group, while Ibrutinib 1799, Nelarabine 1814, and Wee1 Inhibitor 1046 demonstrated higher IC_50_ values in the WT group.

Moreover, only the stromal score demonstrated a significant difference between the MUT and WT groups, with the WT group exhibiting a higher stromal score (*p* < 0.05) (Figure 2E).

### 3.3. Analysis Results of Differences in Immune Infiltration, Anti-Tumor Immune Status and Drug Sensitivity Under Stratification of Key Clinical Features

To assess the universality and robustness of the observed differences between *BRCA1/2* mutant (MUT) and wild-type (WT) TNBC, we conducted a stratified analysis of key clinical variables such as age, race, cancer stage, and menopausal status. The immune infiltration pattern remained consistent among subgroups. At most clinical levels, the infiltration levels of eosinophils, neutrophils, CD56bright natural killer cells and type 17 T helper cells in the WT group were significantly higher than those in the MUT group (Supplementary Figure S1). For instance, in patients classified as White, the infiltration of eosinophils (*p* = 0.030) and neutrophils (*p* = 0.048) was elevated in the WT group. There was an exception among premenopausal patients: the infiltration of type 2 T helper cells was higher in the MUT group (*p* = 0.035). This overall trend confirms that the immunodeficient microenvironment in *BRCA1/2* mutant tumors is a robust phenotype that is not driven by clinical confounding factors. The anti-cancer immune status shows subgroup-specific differences in the recruitment steps. Stratified analysis of the tumor immune cycle revealed that the differences were mainly concentrated in step 4 (recruitment of immune cells) and varied according to clinical conditions (Supplementary Figure S2). For instance, in patients under 50 years old, the recruitment score of Th22 cells in the MUT group was higher (*p* = 0.041), while, in premenopausal patients, the recruitment score of dendritic cells in the WT group was higher (*p* = 0.046). These findings suggest that *BRCA1/2* mutations may damage specific immune recruitment pathways depending on the clinical background of the patients. The results of the drug sensitivity difference analysis indicate that. The IC_50_ differences observed between the MUT group and the WT group throughout the cohort generally remained unchanged in the clinical subgroups. Among patients aged ≥ 50 years, the MUT group showed higher resistance to AZD8186_1918, Acetalax_1804 and GNE-317_1926. Conversely, in stage II patients, the WT group was more resistant to Ibrutinib_1799 and Nelarabine_1814 (Supplementary Figure S3). These results suggest that, although the *BRCA1/2* mutation status is a key determinant of drug response, its effect can be fine-tuned through clinical factors such as age and disease stage.

### 3.4. The Functions and Enrichment Pathways of 268 DE-EVRGs

**Figure 3:**
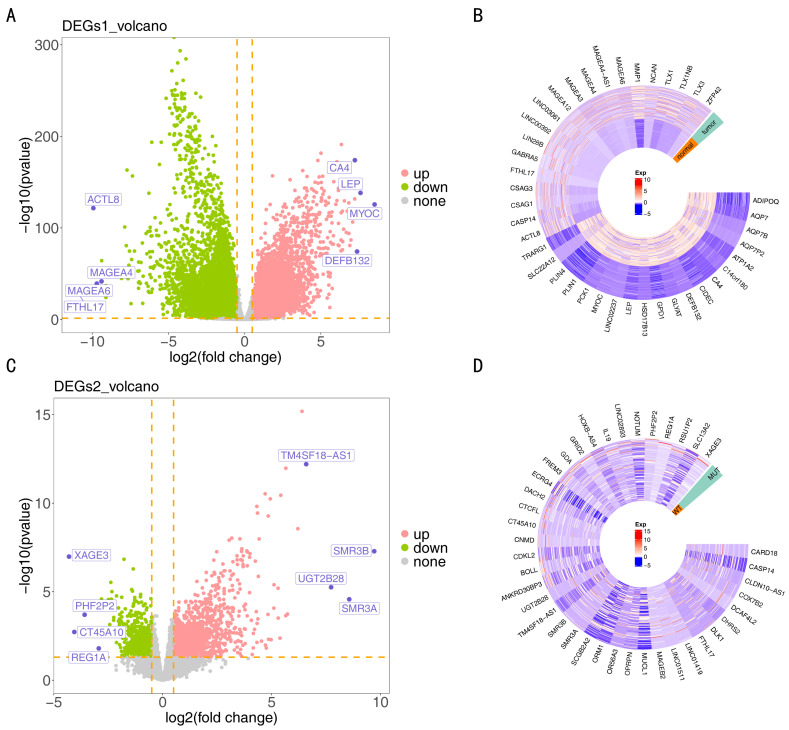
Identification of differentially expressed genes. Volcano plots (**A**) and heatmap (**B**) showed differentially expressed genes 1(DEGs1) between triple-negative breast cancer (TNBC) group and control group. Volcano plots (**C**) and heatmap (**D**) showed differentially expressed genes 2 (DEGs2) between the MUT group and WT group. DEGs, differentially expressed genes.

**Figure 4:**
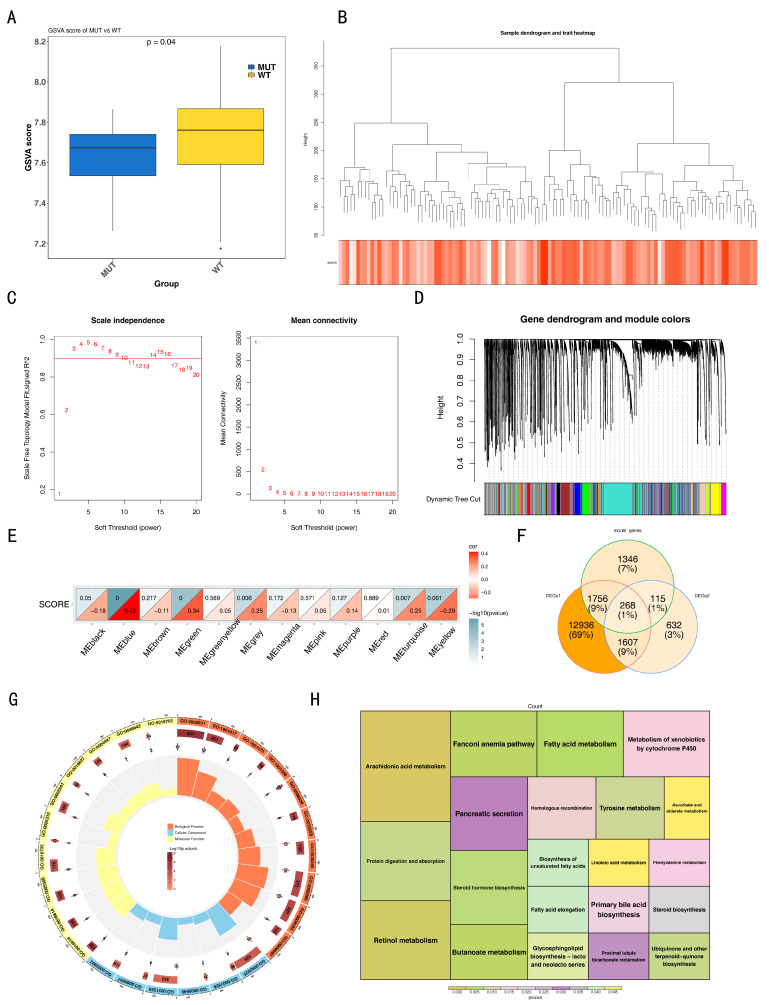
Identification and function analysis of extracellular vesicles related genes (EVRGs). (**A**) Differences in EVRG scores between mutation (MUT) and wild-type (WT) group. (**B**) In the weighted gene co-expression network analysis (WGCNA) analysis, no outlier samples were detected. (**C**) Soft threshold distribution.The horizontal axes all represent the weight parameter power value. The vertical axis of the left graph is scale-free fit index, that is, signed R2. The higher the square of the correlation coefficient, the closer the network is to the scale-free distribution. The vertical axis of the right graph represents the mean of the adjacency function of all genes in the corresponding gene module. (**D**) Co-expression matrix. (**E**) Modules correlated with EVRG scores. (**F**) Overlapped genes by differentially expressed genes 1 and 2 (DEGs1, DEGs2) and key module genes were EVRGs. Gene Ontology (GO) analysis (**G**) and Kyoto Encyclopedia of Genes and Genomes (KEGG) analysis (**H**) were performed to explore the function of EVRGs.

Differential expression analysis identified 16,567 DEGs (7170 upregulated and 9397 downregulated) between TNBC and control samples in TCGA-TNBC (Figure 3A,B). Additionally, 2622 DEGs (1902 upregulated and 720 downregulated) were identified between the MUT and WT samples in TCGA-TNBC (Figure 3C,D). Moreover, the EVRG score was calculated and found to be substantially higher in the WT group (*p* = 0.04) (Figure 4A). In the WGCNA, no outlier samples were chosen in either the MUT or WT group (Figure 4B). Subsequently, a power value of 4 was determined, which exceeded the red line (R2 = 0.9) for the first time, whereas the mean connectivity approached 0 (Figure 4C). Following this, a co-expression matrix was established, revealing 12 gene modules displayed in different colors (Figure 4D). Among these, MEblue (cor = 0.42, *p* < 0.001) was strongly linked to the EVRG score (Figure 4E). Consequently, 3485 genes from MEblue were deemed key module genes. By overlapping 16,567 DEGs1, 2622 DEGs2, and 3485 key module genes, 268 DE-EVRGs were identified (Figure 4F). Functional enrichment analysis indicated that these 268 DE-EVRGs were substantially enriched in 395 GO terms (6 CCs, 59 MFs, and 330 BPs) and 21 KEGG pathways. The significant GO terms included “fatty acid metabolic process”, “collagen-containing extracellular matrix”, and “DNA-binding transcription activator activity”, etc. (Figure 4G). The significant KEGG pathways included the “arachidonic acid metabolism”, “retinol metabolism”, and “Fanconi anemia pathway”, etc. (Figure 4H).

### 3.5. PLA2G5 Was Identified as Biomarker of TNBC

**Figure 5:**
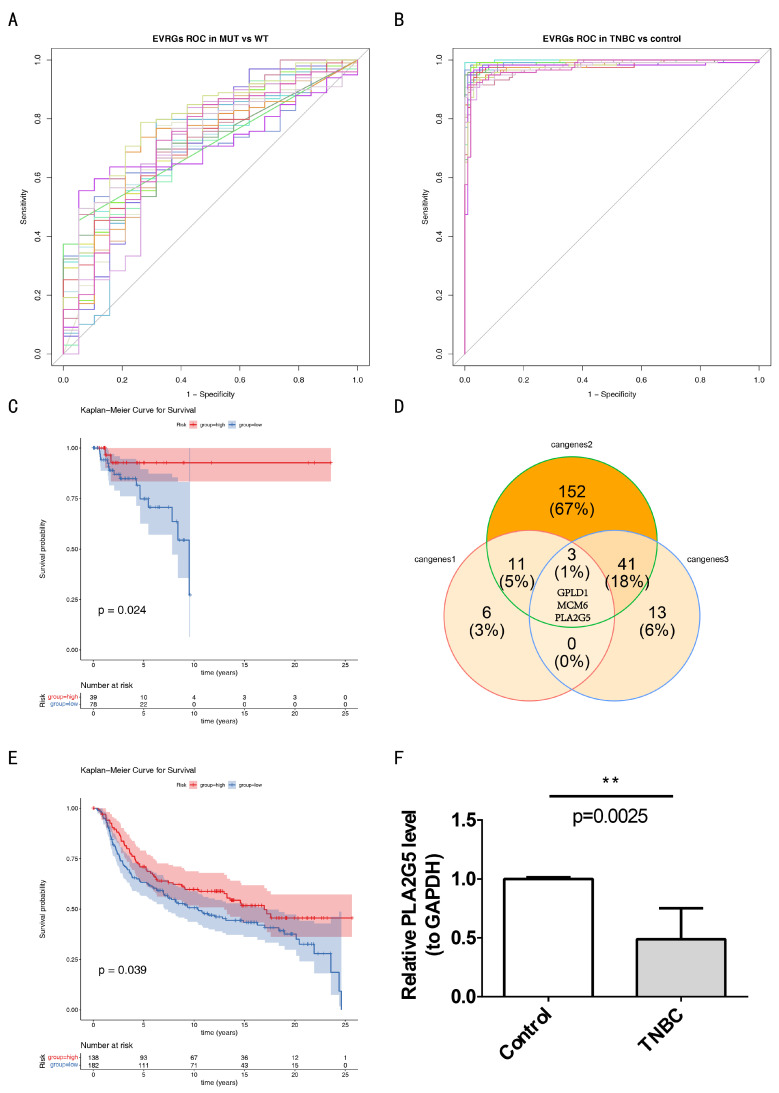
Identification of *PLA2G5* as biomarker of triple-negative breast cancer (TNBC). (**A**) Receiver operating characteristic (ROC) curve to detect candidate genes 1 between mutation (MUT) and wild-type (WT) samples. (**B**) ROC curve to detect candidate genes 2 between TNBC and control samples. (**C**) Kaplan-Meier (K-M) curve of *PLA2G5* showed a significant survival difference between high and low expression groups. (**D**) Three key genes were identified. (**E**) K-M curve of *PLA2G5* in validation cohort. (**F**) reverse transcription quantitative polymerase chain reaction (RT-qPCR) showed expression of *PLA2G5* in TNBC samples. ** indicates *p* < 0.01.

Based on the ROC analysis of the 268 DE-EVRGs, 20 candidate genes 1 were identified as having an excellent ability to distinguish between MUT and WT samples, with an AUC > 0.7 (Figure 5A). Similarly, 206 candidate genes 2 demonstrated a strong ability to differentiate between TNBC and control samples, with an AUC > 0.7 (Figure 5B). Furthermore, 57 candidate genes 3 exhibited a substantial survival difference between the high- and low-expression groups, as shown by the K-M curve (*p* < 0.05). For example, in the case of *PLA2G5*, the high-expression group had a higher survival probability and longer survival time (*p* = 0.024) (Figure 5C). By overlapping sets of 20, 206, and 57 candidate genes 1–3, three key genes (*GPLD1*, *MCM6*, and *PLA2G5*) were identified (Figure 5D). In the validation cohort, only the high expression group of *PLA2G5* showed a significantly higher survival probability (*p* = 0.039), leading to the identification of *PLA2G5* as a biomarker for TNBC (Figure 5E). RT-qPCR showed that the expression of *PLA2G5* (*p* = 0.0025) was significantly lower in TNBC samples (Figure 5F).

### 3.6. Verification of the Independent Prognostic Value of Biomarkers and Correction Results for Clinical Confounding Factors

The verification results of the independent prognostic value of *PLA2G5* showed that *PLA2G5* was a protective factor for TNBC patients in the univariate Cox regression analysis (HR = 0.3578, 90% CI: 0.1397–0.9165, *p* = 0.07) (Supplementary Figure S4A) and passed the Schoenfeld residual test (*p* > 0.05). Meanwhile, cancer stage and pathological M/N/T stage were risk factors, and radiotherapy treatment was a protective factor (Supplementary Figure S4B); After correction by the multivariate Cox regression model, *PLA2G5* remained an independent protective factor for TNBC (HR = 0.2135, 90% CI: 0.0748–0.6094, *p* = 0.0154), cancer stage (*p* < 0.001) (Supplementary Figure S5A), pathological N stage (*p* = 0.0275), and radiotherapy treatment (*p* < 0.001) were also independent prognostic factors. The overall model and all variables met the PH hypothesis (*p* > 0.05) (Supplementary Figure S5B).

### 3.7. Functional and Localization Insights of PLA2G5

**Figure 6:**
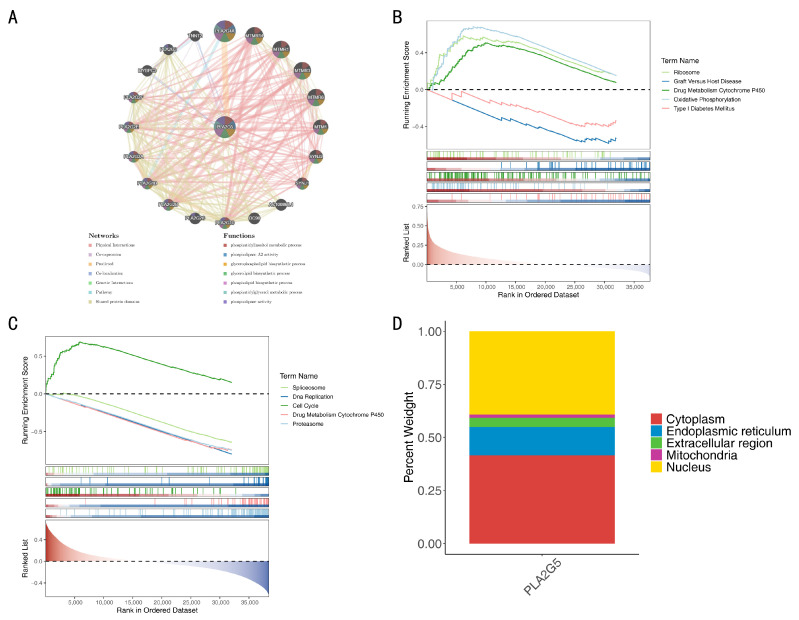
Function and localization of *PLA2G5*. (**A**) GeneMANIA network. (**B**) GESA analysis between mutation (MUT) and wild-type (WT) group. (**C**) Gene set enrichment analysis (GSEA) analysis between triple-negative breast cancer (TNBC) group and control group. (**D**) Subcellular localization analysis of *PLA2G5*.

Using the GeneMANIA database, a search was conducted to identify 20 functionally related genes (e.g., *PLA2G4A* and *MTMR14*) with *PLA2G5*. Subsequently, a GGI network was constructed, highlighting its involvement in various functions, such as the phosphatidylinositol metabolic process and phospholipase A2 activity (Figure 6A). Furthermore, in the comparison between MUT and WT groups, *PLA2G5* was found to be significantly enriched in 10 KEGG pathways, including “ribosome”, “graft Versus Host disease”, “drug metabolism cytochrome P450”, “oxidative phosphorylation”, and “type I diabetes mellitus” among others (Figure 6B). Similarly, in the comparison between TNBC and control samples, *PLA2G5* exhibited significant enrichment in 80 KEGG pathways, including “spliceosome”, “DNA replication”, “cell cycle”, “drug metabolism cytochrome P450”, and “proteasome” among others (Figure 6C). Notably, the “drug metabolism cytochrome P450” pathway, in which *PLA2G5* was significantly enriched, may play an important role in TNBC. Subcellular localization analysis via mRNALocater database prediction indicated that *PLA2G5* was predominantly localized in the cytoplasm and nucleus (Figure 6D). Additionally, chromosomal localization analysis revealed that *PLA2G5* is situated on chromosome 1 (Supplementary Figure S6).

### 3.8. The Regulation Networks of PLA2G5 Were Helpful for Exploring the Molecular Mechanism of TNBC

**Figure 7:**
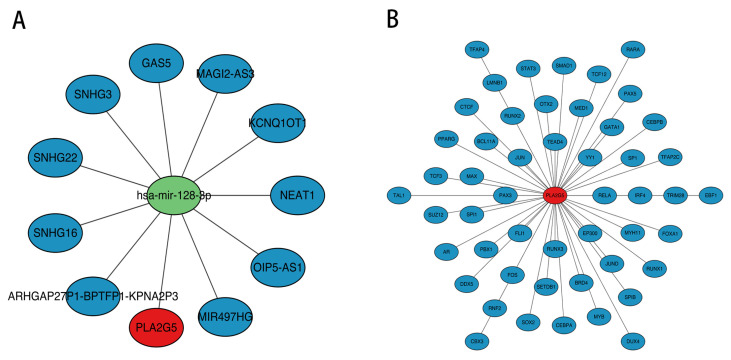
The regulation networks of *PLA2G*. (**A**) lncRNA-miRNA-mRNA network. Red nodes represent key genes, green nodes represent mirnas, and blue nodes represent lncrnas. (**B**) TF-biomarker network. Red nodes represent key genes, and blue nodes represent the TFs predicted by key genes.

Through prediction and integration, we identified one key miRNA (hsa-mir-128-3p) targeting *PLA2G5* and ten key lncRNAs (eg. *SNHG16*, *GAS5*, etc.) targeting hsa-mir-128-3p. This led to the construction of a comprehensive lncRNA-miRNA-mRNA regulatory network, which comprises 12 nodes (10 key lncRNAs, 1 core miRNA [hsa-mir-128-3p], and 1 target mRNA [*PLA2G5*]) and 11 edges that reflect the direct targeting relationships between the lncRNAs and hsa-mir-128-3p, as well as between hsa-mir-128-3p and *PLA2G5* (Figure 7A). Fifty TFs targeting *PLA2G5* were predicted using the hTFtarget database, including RELA, RUNX3, and EP300. A TF-biomarker network was constructed, including RELA-PLA2G5 and RUNX3-PLA2G5 (Figure 7B).

### 3.9. Drug Assessment of Biomarkers

**Figure 8:**
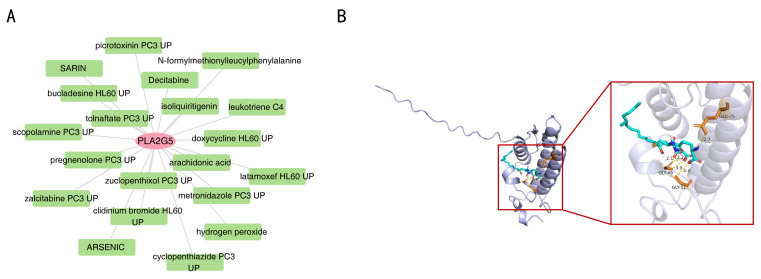
The drug assessment of *PLA2G5*. (**A**) A drug-biomarker network of *PLA2G5*. (**B**) Molecular docking analysis showed that leukotriene C4 bound to PLA2G5.

In the search for potential targeted therapies, 20 drugs, such as Decitabine and leukotriene C4, were identified as targeting *PLA2G5*, leading to the establishment of a drug-biomarker network (Figure 8A). Notably, leukotriene C4 demonstrated a high interaction score with PLA2G5, which prompted its selection for molecular docking analysis. The docking results revealed that leukotriene C4 and *PLA2G5* achieved a favorable binding energy of -7.2 kcal/mol, with critical interactions occurring at residues GLU-75, GLY-49, and GLY-51, among others (Figure 8B). These computational findings suggest that leukotriene C4 may have a potential therapeutic value by targeting *PLA2G5* in TNBC, which warrants further experimental validation.

## 4. Discussion

TNBC is the subtype with the worst prognosis in breast cancer, and the clinical dilemma of strong heterogeneity and limited treatment methods has not been completely improved. BRCA1/2 mutation is a key molecular feature in TNBC, and its regulatory effect on tumor immune microenvironment, drug response and prognosis has been a research hotspot [29,30]. Based on the TCGA-TNBC cohort, this study focused on the association of *BRCA1/2* mutation status with extracellular vesicle (EV)-related biomarkers through multidimensional analysis. We confirmed that there are significant and robust differences in immune infiltration, anti-tumor immune status and drug sensitivity between *BRCA1/2* mutant group (MUT) and wild-type (WT) TNBC. Ev-related gene *PLA2G5*, as a key biomarker, has prognostic value independent of clinical confounding factors, and has potential regulatory association with *BRCA1/2* mutation status. Its stable targeting combination with leukotriene C4 provides a new direction for the precise treatment of TNBC.

There were 17 cases (100%) in the MUT group with gene mutations, among which the mutational frequency of the *TP53* gene was the highest. A total of 92 cases (93.48%) in the WT group had gene mutations, and the *TP53* gene mutational frequency was the highest in the WT group. In conclusion, the *TP53* mutational frequency was the highest in both groups. *TP53* mutation occurs in all types of BC, but the mutation rate in TNBC can be as high as 80% [31,32]. *TP53* is a pivotal gene that encodes tumor suppressor proteins [33]. Inactivated *TP53* may lead to increased instability of tumor cells and promote malignant transformation and metastasis and considered to be related to chemotherapy insensitivity. TNBC with *TP53* mutations was found to have worse overall survival (OS) and a younger age of onset [34]. Different BC subtypes exhibit distinct *TP53* mutation spectrum. For patients with g*BRCA1/2* and *TP53* co-mutations, the mutation hotspots of *TP53* are different from sporadic BCs. Studies have also found clinical characteristics in patients with germline *BRCA1/2* and *TP53* co-mutations, such as TNBC tendency, high Ki67 value, superior survival outcome, and increased genetic mutations in HR-related genes [35]. However, the mechanism by which BRCA1/2 and *TP53* mutations influence each other is unclear and requires further exploration. Carcinogenic high-level amplification (HLAMP) is a key factor in tumor progression and affects prognosis. Since HLAMPs are very rare in wild-type mammary epithelial cells, they significantly increase with loss of *TP53* function and further increase with loss of *BRCA1/2* function. Thus, the induction of genomic instability in the mammary epithelium results in a gradual increase in the rate of genomic divergence between individual cells [36]. *TP53* is now becoming a potential biomarker and therapeutic target for TNBC, as several compounds reactivating *TP53* mutants have been shown in preclinical models to explore potential anticancer activity [37].

In terms of immune cells, there was a greater proportion of infiltration in the WT group, namely CD56 bright natural killer cells, eosinophils, neutrophils, and type 17 T helper cells. Neutrophils play a critical role in the occurrence, development, and metastasis of cancers, including BC. Peripheral neutrophils accumulate around tumor cells, are driven and recruited by tumor-derived cytokines, and become an important component of TME. They are known as tumor-associated neutrophils (TANs). TANs have the potential of both cancer suppression (N1 phenotype) and cancer promotion (N2 phenotype), which can switch into each other under the influence of many factors [38,39]. It has been revealed that TANs are more common in TNBC compared to other BC types [40]. A meta-analysis that pooled 20 studies found that TANs with high infiltration levels were associated with poor recurrence-free survival (RFS) and OS in multiple cancers [41]. In our study, the WT group had higher TAN infiltration levels than the MUT group did. Does this mean that patients with *BRCA1/2* mutations have worse prognosis? At present, the effect of *BRCA1/2* mutations on TNBC survival and prognosis is controversial [42,43,44]. *BRCA1/2* mutations may affect DNA damage, cell metabolism, and the polarization status of neutrophils, thus affecting tumor development and therapy. However, the detailed mechanisms remain unclear. In contrast, a previous study on inflammatory cells in peripheral blood and BC subtypes found that white blood cells and neutrophil-to-lymphocyte ratio were adverse predictors of TNBC [45]. Neutrophils can migrate to circulating blood in response to inflammation; therefore, it is reasonable to speculate that TANs acquiring tumor-promoting properties may induce tumor metastasis in distant organs and affect tumor prognosis [46].

In the TME of HR-positive BC with *BRCA1/2* mutations, CD8 T cells, natural killer (NK) cells and M2 type macrophages were more abundant, while neutrophils were less abundant, which is consistent with our findings [47]. *BRCA1* mutations were more likely to be TNBC, and *BRCA2* mutations were more likely to be HR+ BCs. This suggests that it may be useful to study the effects of *BRCA1* and *BRCA2* on the dispersion and function of various immune cells in the TME separately. NK cells have innate antitumor effects, not only directly killing tumor cells, but can also rapidly express a variety of cytokines and chemokines, recruit other immune cells, and promote the adaptive immune response of T cells and B cells. Studies have confirmed that NK cells in the TME are linked to the outcome of various cancers, and NK cell therapy has become a new direction of tumor immunotherapy research [48]. In TNBC, NK cells inhibit the invasion of tumor cells through IL-6 mediated urokinase type plasminogen activator (uPA) downregulation, which participates in cancer growth and metastasis [49]. However, the function of NK cells is determined by their activation and maturation state. The characteristics of NK cell subsets in TNBC and their dormant relationships with disease progression are not yet clear. It was observed that more CD56bright NK cells infiltrated the TME of TNBC tumors compared to non-TNBC patients. Immature NK cell subpopulations and immature CD11b-CD27-NK cells can maintain tumor cell stemness and promote tumor progression by upregulating the Wnt signaling pathway in tumor cells, which is associated with poor OS in TNBC patients [50]. Regarding the mechanisms of BRCA1/2 mutations on the distribution and function of NK cells, studies have found that BRCA1/2 mutations can affect their ability to produce granzyme A and perforin in CD8+T cells and CD107a+NK cells by affecting HRR, and this effect is related to the HRD score [51].

In this study, we identified group V phospholipase A2 (*PLA2G5*) as an independent prognostic biomarker of TNBC. It belongs to the group of secretory Ca2+-dependent phospholipase A2 as a member of the PLA2 family and is expressed in cardiomyocytes, lung epithelial cells, gastric fibroblasts, and multiple immune cells (e.g., neutrophils, macrophages, mast cells, and T cells) [52]. There is growing evidence that PLA2 is involved in the genesis and development of tumors and is a potential therapeutic target, of which *PLA2G2* is the most familiar member [53]. *PLA2G5* is closely associated with signal transduction, lipid metabolism, inflammation, and infection. Little research about *PLA2G5* has been conducted on the occurrence and development of cancer, but it has been confirmed to be related to ovarian cancer, prostate cancer, and gliomas [52,54]. Overexpression of *PLA2G5* is associated with poor prognosis in both high-grade and low-grade gliomas. This mechanism may be related to the epithelial-mesenchymal transition (EMT) [55]. PLA2 families mediate the signal transduction process between tumor and endothelial cells by participating in the arachidonic acid cascade signaling pathway, affecting the proliferation, migration, and vascular permeability of endothelial cells56. Isocitrate dehydrogenase 1 mutation status was also correlated with high expression of *PLA2G5*. *PLA2G5* is also involved in immune regulation and plays a pro-inflammatory or anti-inflammatory role in immune diseases. *PLA2G5* is a Th2/M2-inclined sPLA2, and IL-4 can induce *PLA2G5* expression in M2 macrophages and Th2 cells to shift the immune balance to the Th2/M2 state and promote Th2 type immune response [56]. M2-type macrophages suppress T cell immunity by expressing the immunosuppressive molecule PD-L1, which disrupts antitumor immunity and is associated with poor prognosis. However, our study regarded *PLA2G5* as a protective factor for TNBC because the high expression group had better survival prognosis. This paradoxical phenomenon also occurs in other sPLA2s. For example, sPLA2-IIA is a poor prognostic marker for bowel and prostate cancers and is an independent predictor of a good prognosis for gastric cancer. Research has shown that, except for glioblastoma, *PLA2G5* expression is significantly decreased in breast, prostate, lung, brain medulloblastoma, ovarian, and bladder cancers. It has been shown that downregulation of PLA2G5 in cancer cells is controlled by epigenetic mechanisms such as histone deacetylation and DNA methylation [57]. However, it remains unclear why *PLA2G5* exerts opposing effects on carcinogenesis.

GESA revealed that the *PLA2G5* gene was enriched in the “drug metabolism cytochrome P450” pathway, both in terms of MUT vs WT groups and TNBC vs. control groups. Cytochrome P450 reductase (CYPOR) is involved in drug metabolism, hormone synthesis, cholesterol synthesis, fatty acid metabolism, and metabolism of exogenous compounds. CYPOR overexpression induces TAM resistance through the STAT1/c-Myc pathway and may serve as an independent prognostic biomarker for TAM treatment in breast cancer patients [58]. Studies have found that TNBC with high CPYOR expression had shorter RFS, which is helpful in identifying patients in need of stronger intensity adjuvant treatment and follow-up monitoring [59]. Thus, it is a promising prognostic biomarker for TNBC.

We also identified one miRNA, hsa-mir-128-3p, and 50 TFs targeting *PLA2G5*, including *ELA*, RUNX3, and *EP300*. High mir-128-3p levels are correlated with decreased RFS in TNBC [60]. It can suppress the progression of cancer and is recognized as a potential biomarker of TNBC. Runt-related transcription factor 3 (*RUNX*) and *E1A* Binding Protein P300 (*EP300*) are tumor suppressors in breast cancer, especially in TNBC, involved in tumor inhibition, drug resistance, EMT, and stemness regulation, and play an important role in the treatment and prognosis of breast cancer [61,62,63].

In this study, we revealed the differences in the mutation spectrum, immune microenvironment, and drug sensitivity between *BRCA1/2* mutant and WT TNBC via bioinformatics analysis, and *PLA2G5* was identified as a potential prognostic marker. However, this study has several limitations. First, the sample size of the MUT group (*n* = 19) and the WT group (*n* = 99) in the TCGA cohort was seriously unbalanced, which may affect the statistical power and robustness of the conclusions. Secondly, PLA2G5 validation was only based on RT-qPCR and bioinformatics analysis of 10 clinical samples. There was a lack of direct verification of protein levels (such as Western Blot) in cells or tissues, and a lack of stratified analysis of *TP53/BRCA1/2* mutation status of samples. The direct association has not been confirmed by EV isolation, characterization and functional experiments. In addition, drug prediction, such as leukotriene C4, relies only on computational simulations and lacks experiment and in vivo verification. At the same time, there is a lack of *PLA2G5* gene function experiments (knockdown/overexpression) and animal models, and its biological mechanism has not been elucidated. Finally, the regulatory network proposed in this study is completely based on bioinformatics prediction, and its authenticity has not been verified by experiments. The clinical sample size is also limited, and larger independent cohorts are needed to verify its prognostic value.

Future studies should systematically verify the conclusions of this study from the following dimensions. First, multi-omics sequencing and gene editing technology should be integrated to clarify the mutation background, combine with xenograft models and supplement *PLA2G5* protein level verification in TNBC cells and tissues, and fully reveal its biological function. Secondly, EV isolation and functional experiments are needed to clarify whether *PLA2G5* is a key component of EVs involved in tumor communication. Single-cell and spatial transcriptome technology was used to analyze their interaction with the tumor microenvironment. In addition, its prognostic value will be validated in larger prospective clinical cohorts, and the predictive drugs such as leukotriene C4 will be systematically reviewed from molecular docking, cellular function to in vivo efficacy. Finally, prospective clinical studies will be conducted to evaluate the translational potential of *PLA2G5* as a diagnostic marker or therapeutic target to provide a new path for precise diagnosis and treatment of TNBC.

## 5. Conclusions

Our study highlights extracellular vesicles in TNBC and focuses on the mutational landscape of *BRCA1/2* in TNBC. The EV biomarker *PLA2G5* has been suggested to be an independent prognostic factor for TNBC and *BRCA1/2* mutations. The biological pathways of biomarker involvement and regulatory network relationships were obtained to further explore the possible mechanism and theoretical basis of EV gene mutations in TNBC-*BRCA1/2*. These findings require further real-world studies to verify the functions of *PLA2G5* in TNBC.

## Figures and Tables

**Table 1:** Clinical characteristics of mutation (MUT) and wild-type (WT) group in the Cancer Genome Atlas-Triple-Negative Breast Cancer (TCGA-TNBC) cohort.

	Mutation Group	Wild-Type Group
Number	19	99
Stage		
Stage I	3/18 (16.7%)	15/97 (15.5%)
Stage II	12/18 (66.7%)	63/97 (64.9%)
Stage III	3/18 (16.7%)	18/97 (18.6%)
Stage IV	0	1/97 (1.0%)
Race		
White	16/19 (84.2%)	71/93 (71.7%)
African	3/19 (15.8%)	14/93 (14.1%)
Asian	0	8/93 (8.1%)
pathologic M		
M0	19/19 (100%)	92/99 (92.9%)
MX	0	6/99 (6.1%)
M1	0	1/99 (1.0%)
pathologic N		
N0	13/19 (68.4%)	61/99 (61.6%)
N1	4/19 (21.1%)	24/99 (24.2%)
N2	1/19 (5.3%)	8/99 (8.1%)
N3	1/19 (5.3%)	6/99 (6.1%)
pathologic T		
T1	3/19 (15.8%)	64/99 (64.6%)
T2	15/19 (78.9%)	21/99 (21.2%)
T3	1/19 (5.3%)	10/99 (10.1%)
T4	0	3/99 (3.0%)
TX	0	1/99 (1.0%)

## Data Availability

The original data presented in this study are openly available in the TCGA database (https://portal.gdc.cancer.gov/, accessed on 19 January 2024), cBioPortal database (https://www.cbioportal.org/, accessed on 25 January 2024), Molecular Signatures Database (MSigDB, https://www.gsea-msigdb.org/gsea/msigdb, accessed on 25 January 2024), and Supplementary Materials. Further inquiries can be directed to the corresponding authors.

## Supplementary Materials

The following supporting information can be downloaded at: https://www.mdpi.com/article/10.3390/biomedicines14010178/s1, Table S1: The 25 extracellular vesicles related genes (EVRGs) collected from Molecular Signatures Database; Table S2: The information table of hierarchical analysis; Table S3: The sequences of primers used for reverse transcription quantitative polymerase chain reaction (RT-qPCR). Figure S1: Stratified analysis of immune infiltration. The stratification was based on age, race, cancer stage, menopausal status, whether targeted therapy was received, and whether chemotherapy was received. ns indicates insignificant; * indicates *p* < 0.05; ** represents *p* < 0.01; Figure S2: Stratified analysis of differences in key steps of tumor immunity. ns indicates insignificant; * indicates *p* < 0.05; ** represents *p* < 0.01; Figure S3: Stratified analysis of drug sensitivity. They are respectively age, race, cancer stage and menopausal status. ns indicates insignificant; * indicates *p* < 0.05; Figure S4: (A) Single-factor independent prognostic analysis forest plot. The horizontal axis represents the hazard ratio, and the vertical axis represents the significant variables retained by the univariate regression. The HR and the corresponding 90% CI horizontal lines represent the impact of the variable on the outcome. (B) PH test plot of independent prognostic univariate regression variables. The horizontal axis represents time, and the vertical axis represents the Schoenfeld residuals of survivals related prognostic factors. The red dots represent the residual values at different time points, the black solid line indicates the trend line of the residuals, and the dotted line represents the confidence interval. A *p* value greater than 0.05 indicates that there is insufficient evidence to reject the proportional risk hypothesis, that is, the survival-related prognostic factors meet the proportional risk conditions in the model; Figure S5: (A) Factor independent prognostic analysis forest plot. (B) PH test plot of independent prognostic multivariate regression variables; Figure S6: Chromosomal localization analysis of PLA2G5.

## Abbreviations

The following abbreviations are used in this manuscript:

BRCA1/2	Breast cancer susceptibility gene 1 or 2
EVRGs	Extracellular vesicles-related genes
DEG	Differentially expressed genes
EV	Extracellular vesicles
HER-2	Human epidermal growth factor receptor 2
KEGG	Kyoto Encyclopedia of Genes and Genomes
GSEA	Gene Set Enrichment Analysis
GO	Gene Oncology
PLA2G5	Group V Phospholipases A2
PR	Progesterone receptor
ROC	Receiver operating characteristic
TME	Tumor microenvironment
TNBC	Triple-negative breast cancer
WGCNA	Weighted Gene Co-expression Network Analysis
