# Supplementary material for: Mutational Landscape Analysis of BRCA1/2 and Identification of Extracellular-Vesicle-Related Biomarkers in Triple-Negative Breast Cancer
Source: Biomedicines. 2026 Jan 14;14(1):178. doi: 10.3390/biomedicines14010178 (PMC12839138; doi:10.3390/biomedicines14010178)
Supplement: Supplementary file 1 [file biomedicines-14-00178-s001.zip › Supplementary Table S1.pdf]

Supplementary Table S1. The 25 EVRGs collected from Molecular Signatures Database.

**Gene**
ARRDC1
ARRDC4
ATP13A2
ATP9A
CD34
CHMP2A
CHMP3
CHMP6
COPS5
HGS
IFNG
PDCD6IP
PRKN
RAB11A
RAB27A
RAB7A
SDC1
SDC4
SDCBP

SMPD3
SNF8
STAM
TSG101
VPS4A
VPS4B
