# Supplementary material for: Mutational Landscape Analysis of BRCA1/2 and Identification of Extracellular-Vesicle-Related Biomarkers in Triple-Negative Breast Cancer
Source: Biomedicines. 2026 Jan 14;14(1):178. doi: 10.3390/biomedicines14010178 (PMC12839138; doi:10.3390/biomedicines14010178)
Supplement: Supplementary file 1 [file biomedicines-14-00178-s001.zip › Supplementary Table S3.pdf]

Supplementary Table S3. The sequences of primers used for RT-qPCR.

primer	sequence
PLA2G5 F	GTCCTCATCATCGGTCACTCC
PLA2G5 R	ATGAGCAACTTCCTTGGGCA
internal reference -GAPDH F	CGAAGGTGGAGTCAACGGATTT
internal reference -GAPDH R	ATGGGTGGAATCATATTGGAAC
