# Supplementary figures and images for: Mutational Landscape Analysis of BRCA1/2 and Identification of Extracellular-Vesicle-Related Biomarkers in Triple-Negative Breast Cancer
Source: Biomedicines. 2026 Jan 14;14(1):178. doi: 10.3390/biomedicines14010178 (PMC12839138; doi:10.3390/biomedicines14010178)

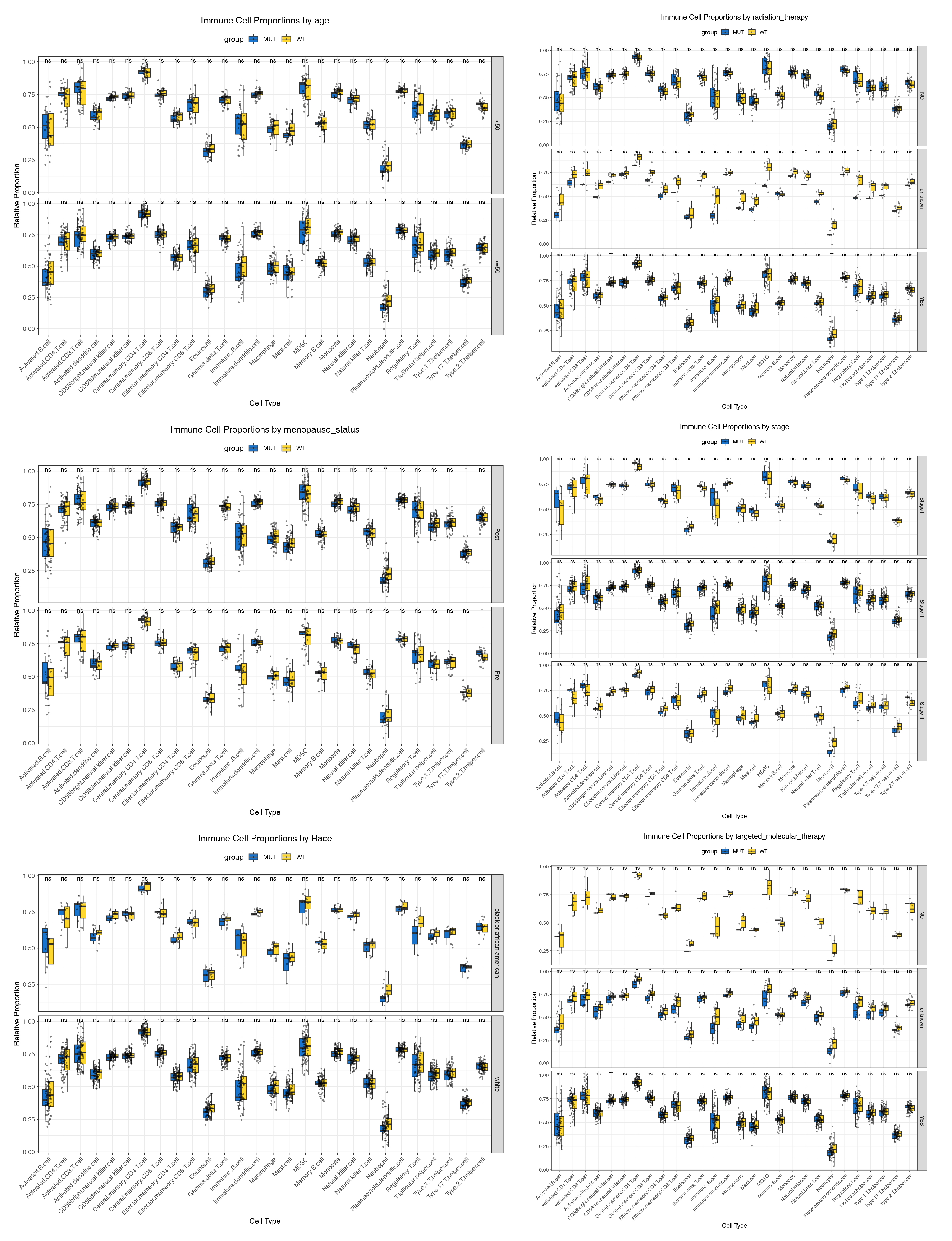

Supplement: Supplementary file 1 [file biomedicines-14-00178-s001.zip › Figure S1.tif]

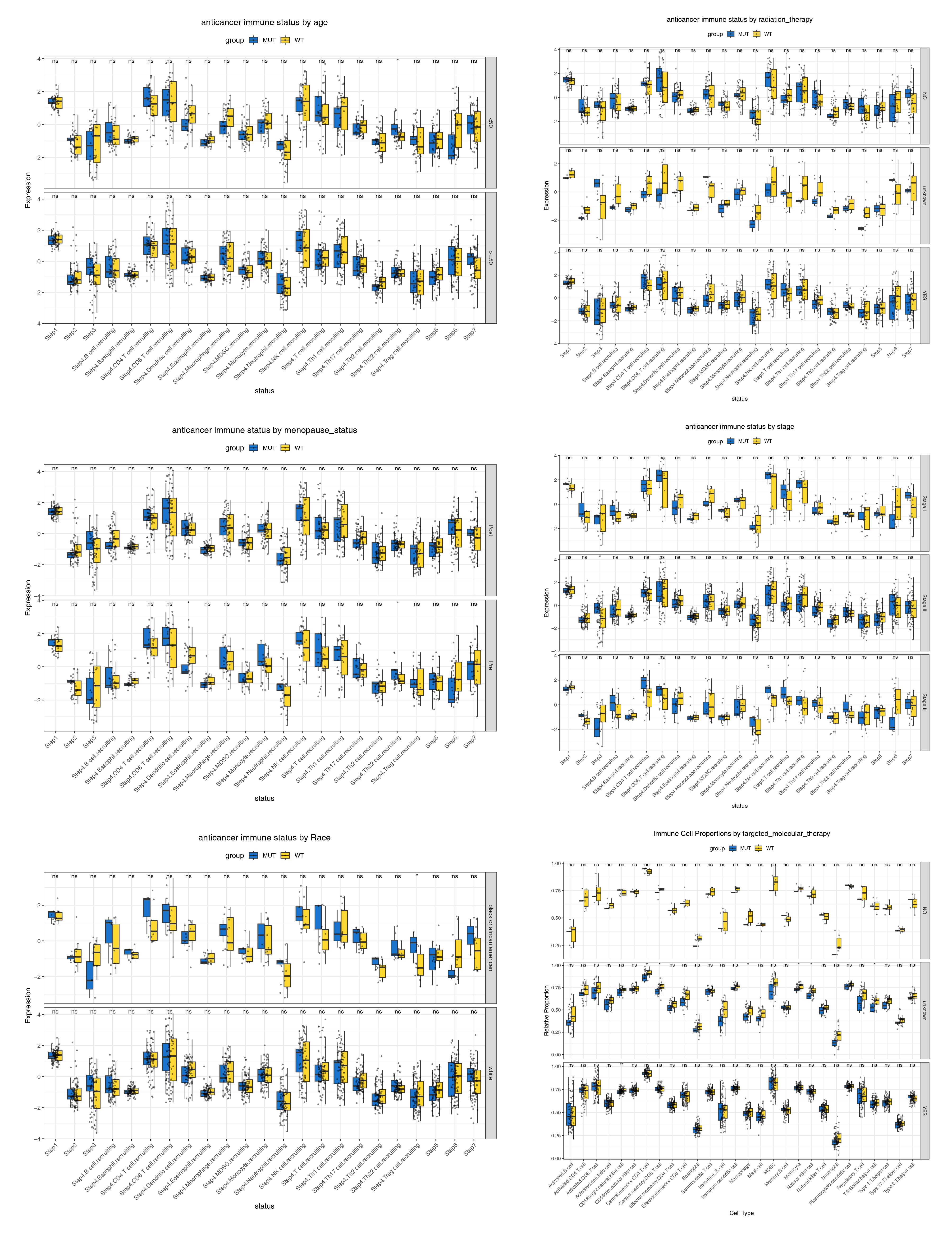

Supplement: Supplementary file 1 [file biomedicines-14-00178-s001.zip › Figure S2.tif]

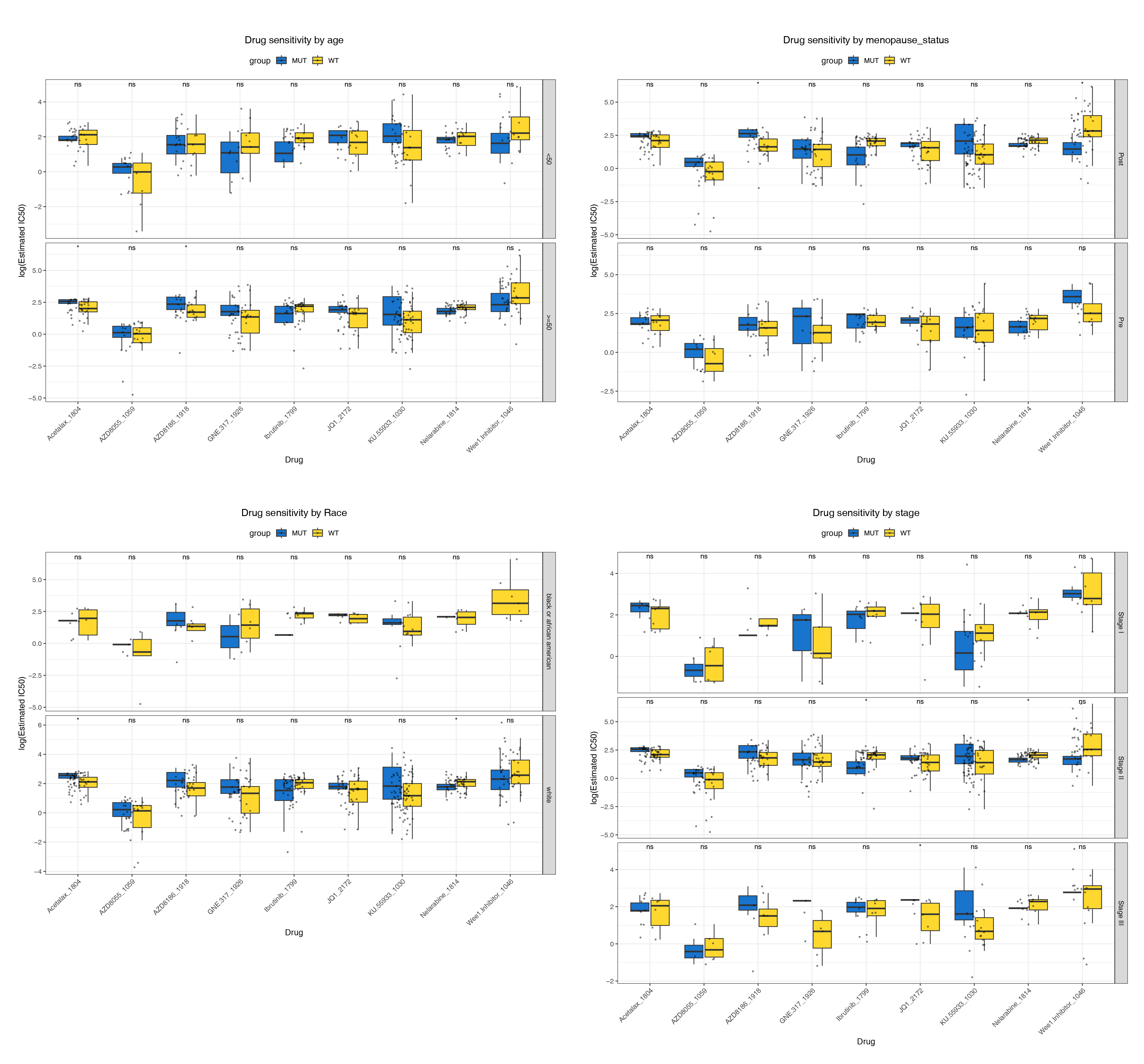

Supplement: Supplementary file 1 [file biomedicines-14-00178-s001.zip › Figure S3.tif]

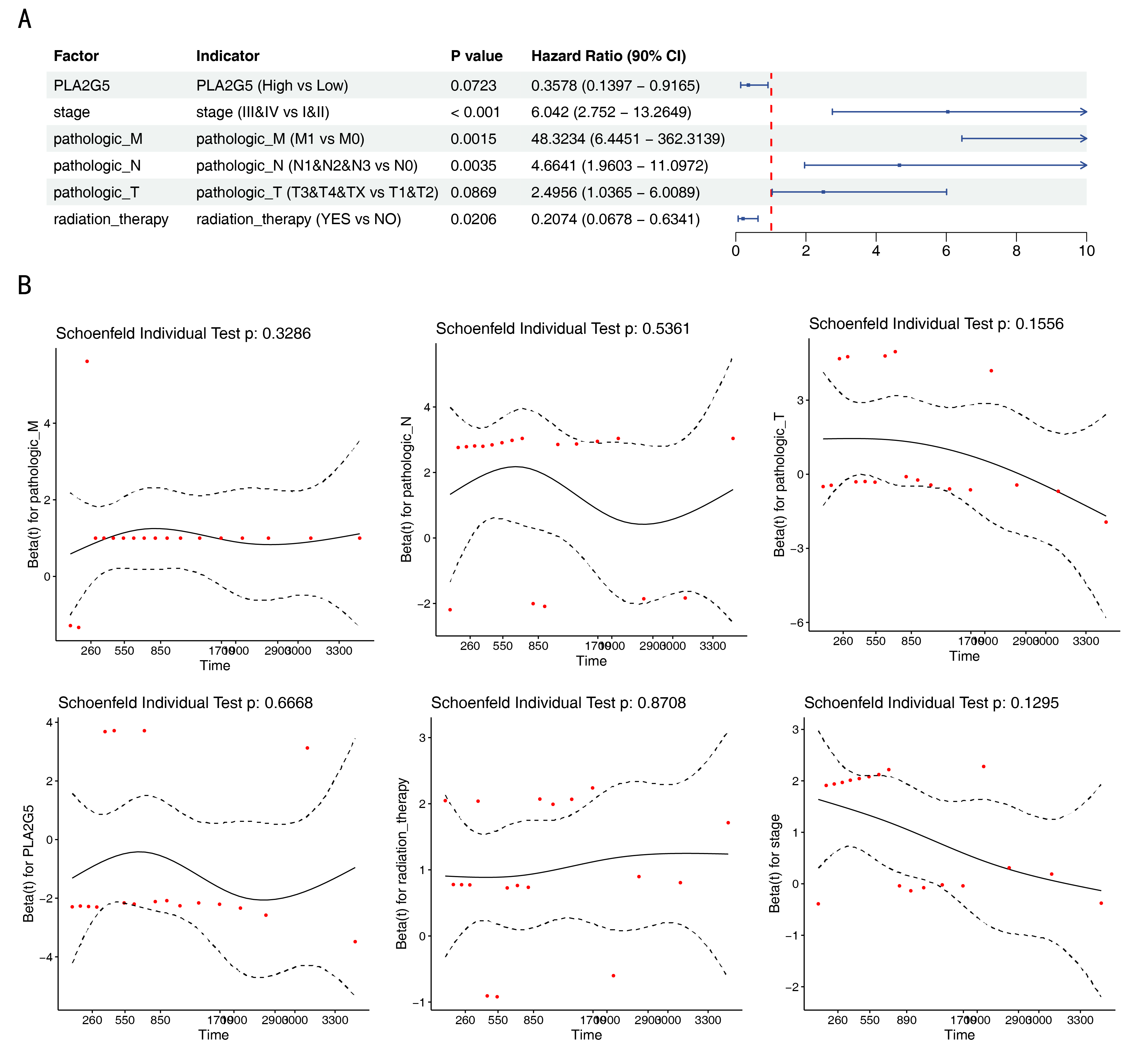

Supplement: Supplementary file 1 [file biomedicines-14-00178-s001.zip › Figure S4.tif]

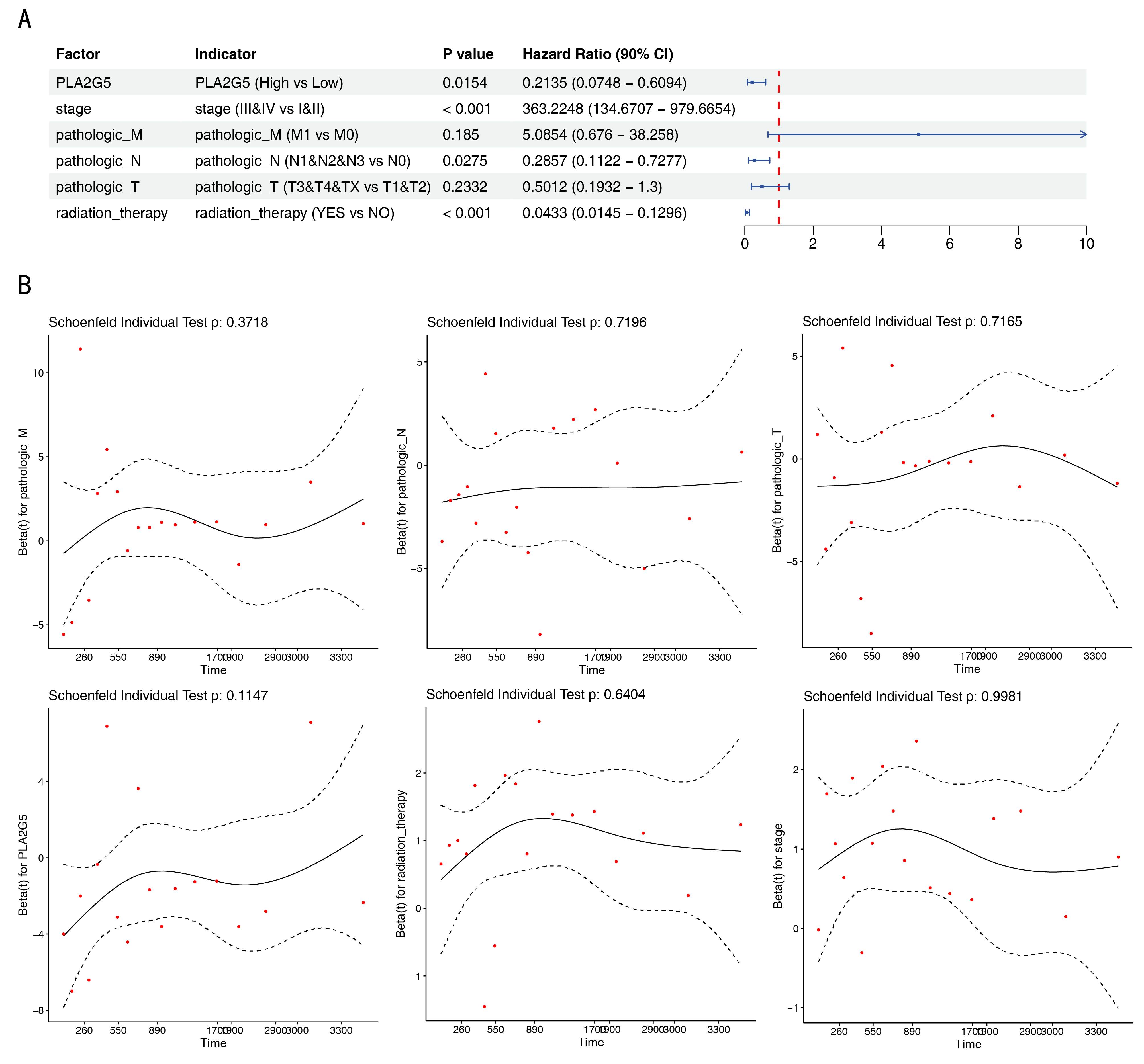

Supplement: Supplementary file 1 [file biomedicines-14-00178-s001.zip › Figure S5.tif]

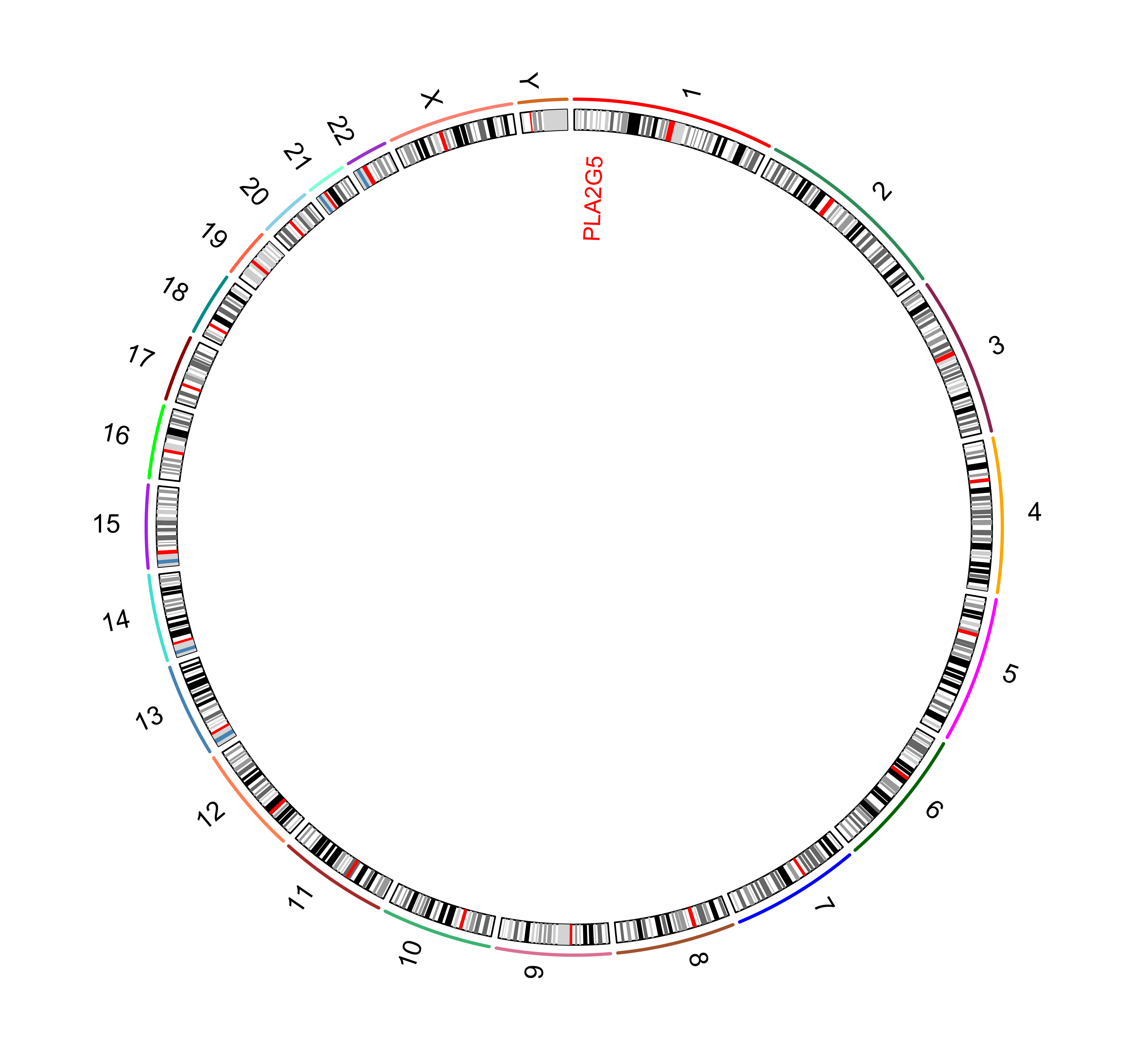

Supplement: Supplementary file 1 [file biomedicines-14-00178-s001.zip › Figure S6.tif]
